# Effect of Keishibukuryogan on Genetic and Dietary Obesity Models

**DOI:** 10.1155/2015/801291

**Published:** 2015-02-22

**Authors:** Fengying Gao, Satoru Yokoyama, Makoto Fujimoto, Koichi Tsuneyama, Ikuo Saiki, Yutaka Shimada, Yoshihiro Hayakawa

**Affiliations:** ^1^Division of Pathogenic Biochemistry, Institute of Natural Medicine, University of Toyama, Toyama 930-0194, Japan; ^2^Department of Japanese Oriental Medicine, Graduate School of Medical and Pharmaceutical Sciences, University of Toyama, Toyama 930-0194, Japan; ^3^Department of Diagnostic Pathology, Faculty of Medicine, University of Toyama, Toyama 930-0194, Japan

## Abstract

Obesity has been recognized as one of the most important risk factors for a variety of chronic diseases, such as diabetes, hypertension/cardiovascular diseases, steatosis/hepatitis, and cancer. Keishibukuryogan (KBG, Gui Zhi Fu Ling Wan in Chinese) is a traditional Chinese/Japanese (Kampo) medicine that has been known to improve blood circulation and is also known for its anti-inflammatory or scavenging effect. In this study, we evaluated the effect of KBG in two distinct rodent models of obesity driven by either a genetic (SHR/NDmcr-cp rat model) or dietary (high-fat diet-induced mouse obesity model) mechanism. Although there was no significant effect on the body composition in either the SHR rat or the DIO mouse models, KBG treatment significantly decreased the serum level of leptin and liver TG level in the DIO mouse, but not in the SHR rat model. Furthermore, a lower fat deposition in liver and a smaller size of adipocytes in white adipose tissue were observed in the DIO mice treated with KBG. Importantly, we further found downregulation of genes involved in lipid metabolism in the KBG-treated liver, along with decreased liver TG and cholesterol level. Our present data experimentally support in fact that KBG can be an attractive Kampo medicine to improve obese status through a regulation of systemic leptin level and/or lipid metabolism.

## 1. Introduction

Obesity has been recognized as one of the most important risk factors for a variety of chronic diseases, such as diabetes, hypertension/cardiovascular diseases, steatosis/hepatitis, and cancer [1, 2]. Accumulating evidence indicates that the pathogenesis of obesity-related metabolic dysfunction involves the development of a systemic low-grade inflammatory state and a deregulated lipid metabolism. Furthermore, it has become apparent that adipose tissue can be a source of secreted regulatory proteins called adipokine which act as modulators of metabolic and immunological processes [3].

Among those, an adipokine leptin is known as the product of the obese gene (ob) that was identified in spontaneous obese ob/ob mice [4]. Leptin regulates feeding behavior; therefore rodents genetically lacking leptin or its receptor show hyperphagia and subsequently develop obesity and insulin resistance. Although leptin itself showed an improving effect in metabolic dysfunction, the blood leptin levels have been known to correlate with adipose tissue mass, and such pathologically elevated levels of leptin did not induce anorexia in obese humans and rodents, therefore suggesting that leptin resistance can be common in obesity [5].

Traditional Chinese/Japanese medicine has a long history and has contributed to the prevention and treatment of various diseases. Keishibukuryogan (KBG, Gui Zhi Fu Ling Wan in Chinese) is one of the formulations in the ancient Chinese medicine and is composed of five crude drugs:* Cinnamomi Cortex*,* Poria cocos*,* Moutan Cortex*,* Persicae Semen*, and* Paeoniae Radix*. While KBG has been originally used for the treatment of gynecological diseases, it has also been used for the treatment of blood hemorheology, platelet aggregation, and inflammation. Previous preclinical studies revealed that KBG inhibits the development of atherosclerosis and prevents nonalcoholic steatohepatitis in cholesterol-fed rabbits [6, 7], improves endothelial function in hypertensive rats [8], shows protective effects on vascular injury in diabetic rats [9], and reduces oxidative stress by hyperglycemia in WBN/Kob rats [8]. Importantly, even in a clinical study, long-term KBG treatment improved vascular endothelial function and resulted in the prevention of atherosclerosis [10]. Collectively, the preclinical and clinical evidence strongly suggest that KBG can be generally useful for the prevention and treatment of tissue damage caused by metabolic dysfunction through its anti-inflammatory and/or antioxidative effect.

In order to explore the effect of KBG on obesity, we tested the therapeutic effect of KBG on two preclinical rodent obesity models driven by either a genetic or dietary mechanism. While the SHR/NDmcr-cp (SHR) rat model has been known to develop metabolic disorders due to its genetic deficiency in leptin receptor [11], the high-fat diet-induced obesity (DIO) model in C57BL/6 mice has been widely recognized as an obesity model driven by a dietary mechanism [12, 13]. Although there was no obvious effect in the body composition of obese animals, KBG treatment significantly decreased the serum level of leptin and liver lipid content in the DIO mouse, but not in the SHR rat model. In concert with its lipid-lowering effect, KBG treatment improved steatosis and adipocyte enlargement in the DIO mouse and further downregulated the expression of genes involved in lipid metabolism (PPAR*γ*, SERBP1) in the KBG-treated liver. Our presented data experimentally support that KBG can be an attractive Chinese/Japanese traditional medicine to improve obese status through the regulation of systemic leptin levels and/or liver lipid metabolism.

## 2. Materials and Methods

### 2.1. Preparation of Keishibukuryogan (KBG)

**Figure 1:**
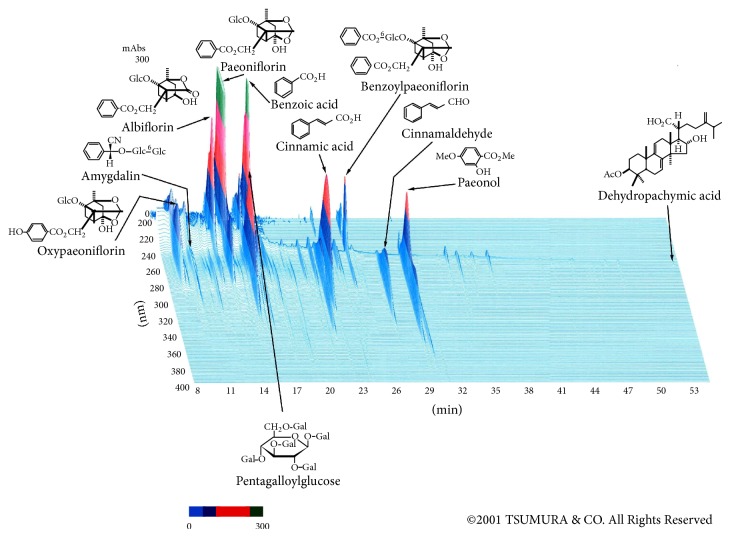
Analysis by three-dimensional HPLC of major chemical compounds included in keishibukuryogan extract.

The extract of KBG was kindly provided by TSUMURA & Co. (TJ-25, Tokyo). The prescription of crude drugs constituting KGB (Table 1) was added to water and extracted at 100°C for 1 hr. The water extract was filtered and spray-dried to obtain a dry extract powder. The 3D-HPLC chart of the KBG extract provided by TSUMURA & Co. is shown as in Figure 1 for quality reference.

### 2.2. Animal Experiments

SHR/NDmcr-cp (SHR) rats (16 weeks old, male) and C57BL/6J mice (5 weeks old, male) were purchased from Japan SLC Inc. (Hamamatsu, Japan). All experiments were approved by and performed according to the Guidelines of the Care and Use of Laboratory Animals of the University of Toyama. For the spontaneously developing genetic obesity model, the SHR rats were housed for 9 weeks feeding on normal chow (Labo MR Stock, Nosan, Yokohama, Japan) before being subjected to KBG treatment. A group of SHR rats was administered KBG (orally with gavage, daily at 500 mg/kg dose) or control water for 8 weeks and sacrificed to collect tissue and serum samples. For the diet-induced obesity (DIO) mouse model, a group of C57BL/6J mice were fed with high-fat diet (HFD, D12492, Research Diets Inc., NJ, USA) for 10 weeks to develop chronic obesity. A separate group of mice was fed a normal diet (ND, D12450B, Research Diets Inc.) as a control. After 10 weeks of high-fat diet feeding, the DIO mice were divided into two groups with similar average of body weight and treated with either KBG (orally with gavage, daily at 500 mg/kg dose) or control water for 12 weeks. Upon 8 weeks' treatment with KBG or water, an interim blood sample collection was conducted and then all mice were given normal chow (Labo MR Stock) for another 4 weeks. On termination of the experiment, blood and tissue samples were collected. Body weight and food intake were monitored weekly.

### 2.3. Serum Measurements

Serum leptin or insulin levels were determined by using a specific enzyme-linked immunosorbent assay (ELISA) according to the manufacturer's instruction. The ELISA kit for rat and mouse leptin (Lbis Leptin-Rat kit or Lbis Leptin-Mouse kit) and rat insulin kit (Lbis insulin-Rat kit) were purchased from Shibayagi Co. Ltd. (Shibukawa, Japan.) kit. The mouse insulin ELISA kit was purchased from Morinaga & Co. (Yokohama, Japan).

### 2.4. Liver Lipid Content Measurements

The liver tissues were weighed and homogenized in sodium chloride buffer and then the homogenates were extracted with 5 mL of chloroform and methanol (2 : 1, vol/vol) [14]. The chloroform layers were dried and then triglyceride (TG), total cholesterol (cholesterol), and free fatty acids (FFA) were measured by using LabAssay Cholesterol kit, LabAssay triglyceride kit, and LabAssay NEEA kit (Wako Chemical, Osaka, Japan).

### 2.5. Histological Analysis

Liver and epididymal adipose tissues were collected upon termination of the experiments and immediately fixed with 4% PFA for 1-2 days. The fixed tissue sample was then sliced sequentially into sections 3–5 mm in thickness. Representative sections of the liver or fat tissue 2-3 mm thick were selected and embedded in paraffin for routine histopathological analysis with hematoxylin and eosin (H&E) staining.

### 2.6. Real-Time RT-PCR for Quantitative Assessment of mRNA Expression

Total RNAs were prepared using the RNeasy Plus Mini kit (QIAGEN, Hilden, Germany). The expression level of targeted mRNAs was normalized to m*Gapdh* mRNA by using One Step SYBR PrimeScript RT-PCR kit II (Takara, Kyoto, Japan). The primers used in this experiment are listed in Table 2.

### 2.7. Statistics

Statistical analysis was performed with JMP (SAS Institute Japan, Tokyo). Data were expressed as mean ± SEM. One-way ANOVA followed by Dunnett's test was used to determine the statistical differences among groups.

## 3. Results

### 3.1. Effect of KBG on Body Weight Changes and Tissue Weight in Obesity Models

**Figure 2:**
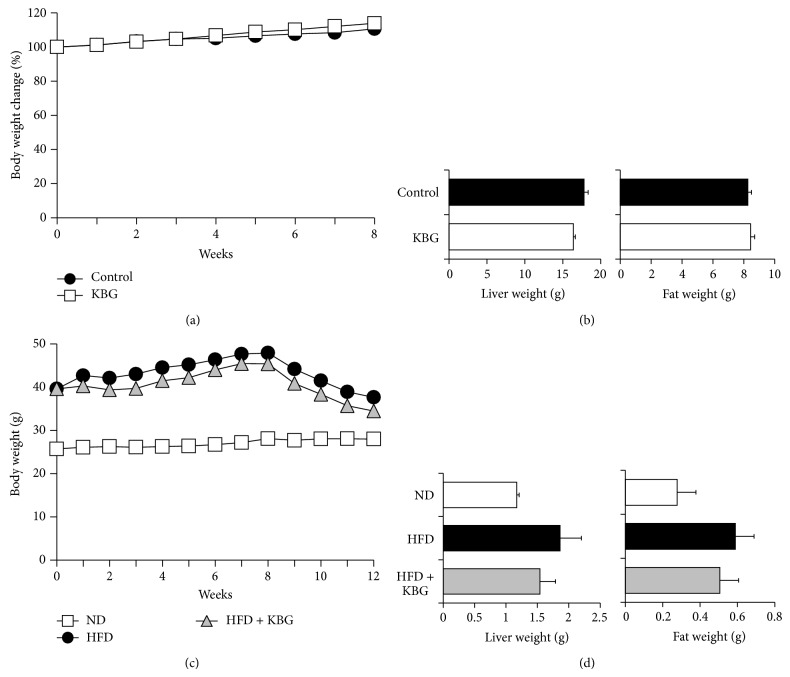
Effect of KBG on body weight changes and tissue weight in obesity models. (a, b) SHR rats were administered with control water or KBG (500 mg/kg, p.o., daily) for 8 weeks. Body weight changes (a) or tissue weight (b, liver and fat, at 8 weeks after daily treatment) of SHR rats is shown. (c, d) C57BL/6 mice were fed with normal diet (ND) or high-fat diet (HFD) for 10 weeks and then administered with control water or KBG (500 mg/kg, p.o., daily) for 12 weeks under the same feeding condition. After 8 weeks of control or KBG treatment, mice fed with HFD were changed to ND until the termination of experiment with maintaining KBG treatment for another 4 weeks. Body weight changes (a) or tissue weight (b, liver and fat at 12 weeks after daily treatment) of DIO mouse is shown. Data are mean ± SEM (*n* = 7–15).

In order to examine the therapeutic efficacy of KBG in genetic or dietary obesity models, we employed an SHR rat model or a DIO mouse model, respectively. As shown in Figures 2(a) and 2(b), KBG treatment did not affect either body weight or tissue weight (liver and epididymal fat) in the SHR rat model. In the DIO mouse model, C57BL/6 mice were fed with HFD for 8 weeks before being subjected to KBG treatment and significantly gained body weight, compared with the ND group. Similar to the SHR rat model, KBG treatment did not affect the body weight gain in the chronic DIO mouse model (Figure 2(c), up to 8 weeks). We then tested the efficacy of KBG in combination with diet modification by feeding mice a standard diet for subsequent 4 weeks from 8-week time point. Even in this condition, we did not see any significant effect on either body weight change or tissue weight (liver and epididymal fat) upon termination. Collectively, KBG did show any dynamic efficacy on either body or tissue weight gain in either genetic or dietary obesity model.

### 3.2. Effect of KBG on Serum Levels of Leptin and Insulin in Obesity Models

**Figure 3:**
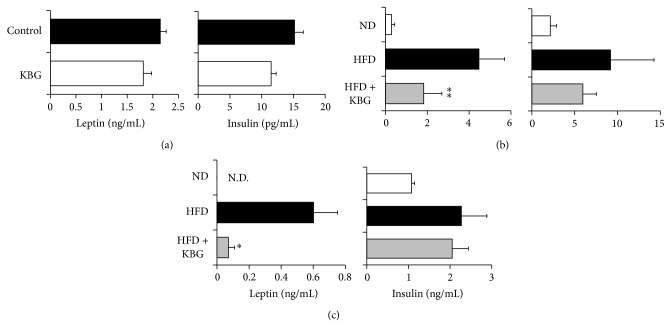
Effect of KBG on serum levels of leptin and insulin in obesity models. (a, b) SHR rats were administered with control water or KBG (500 mg/kg, p.o., daily) for 8 weeks. Serum samples were collected upon the termination and levels of leptin (left) or insulin (right) were measured. (b, c) C57BL/6 mice were fed with normal diet (ND) or high-fat diet (HFD) for 10 weeks and then administered with control water or KBG (500 mg/kg, p.o., daily) for 12 weeks under the same feeding condition. After 8 weeks of control or KBG treatment, mice fed with HFD were changed to ND until the termination of experiment with maintaining KBG treatment for another 4 weeks. Serum samples were collected before the time for food changing (b) or upon the termination (c) and levels of leptin (left) or insulin (right) were measured by using specific ELISA assay. Data are mean ± SEM (*n* = 7–15). ^*^ *P* < 0.005; ^**^ *P* < 0.001.

We then investigated whether KBG treatment affects the obesity-associated serum biomarkers. While KBG treatment did not show any significant effect on the serum levels of leptin in the SHR rat model (Figure 3(a)), DIO mice treated with KBG showed a significantly lower level of serum leptin, both in chronic disease state (Figure 3(b)) and in combination with diet modification (Figure 3(c)). Neither the SHR rats nor the DIO mice treated with KBG showed any significant alteration in their serum insulin level (Figure 3).

### 3.3. Effect of KBG on Lipid Metabolism in Obesity Models

**Figure 4:**
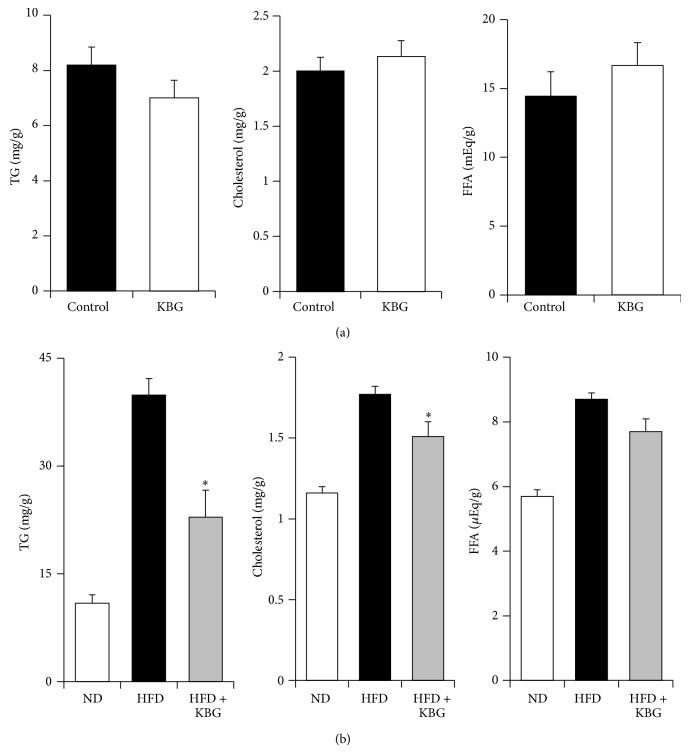
Effect of KBG on liver lipid levels in obesity models. (a) SHR rats were administered with control water or KBG (500 mg/kg, p.o., daily) for 8 weeks. Liver tissue samples were collected upon the termination and levels of triglyceride (TG, left), total cholesterol (cholesterol, middle), and free fatty acid (FAA, right) were measured. (b) C57BL/6 mice were fed with normal diet (ND) or high-fat diet (HFD) for 10 weeks and then administered control water or KBG (500 mg/kg, p.o., daily) for 12 weeks under the same feeding condition. After 8 weeks of control or KBG treatment, mice fed with HFD were changed to ND until the termination of experiment with maintaining KBG treatment for another 4 weeks. Liver tissue samples were collected upon the termination and levels of triglyceride (TG, left), total cholesterol (cholesterol, middle), and free fatty acid (FAA, right) were measured. Data are mean ± SEM (*n* = 7–15). ^*^ *P* < 0.005.

**Figure 5:**
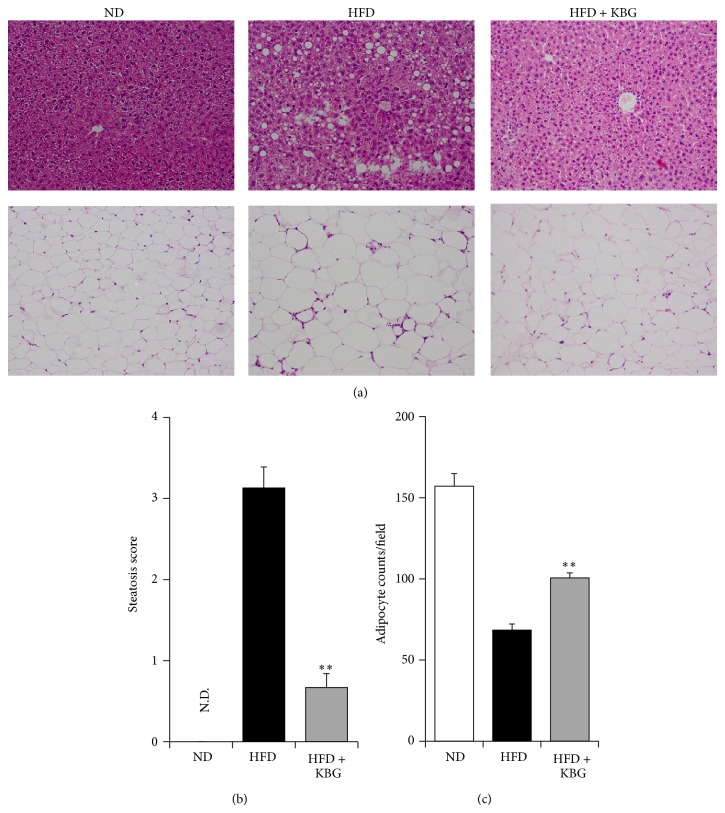
Histopathological evaluation of fat and liver tissue in DIO mice. C57BL/6 mice were fed with normal diet (ND) or high-fat diet (HFD) for 10 weeks and then administered with control water or KBG (500 mg/kg, p.o., daily) for 12 weeks under the same feeding condition. After 8 weeks of control or KBG treatment, mice fed with HFD were changed to ND until the termination of experiment with maintaining KBG treatment for another 4 weeks. (a) Representative images (×200) of H&E staining of liver (upper panels) and white adipose tissue (lower panels) of DIO mice are shown. (b) Steatosis scores of DIO mice. N.D.: not detected. (c) Observed number of adipocyte counts of WAT in DIO mice. Data are mean ± SEM (*n* = 7–15). ^**^ *P* < 0.001.

**Figure 6:**
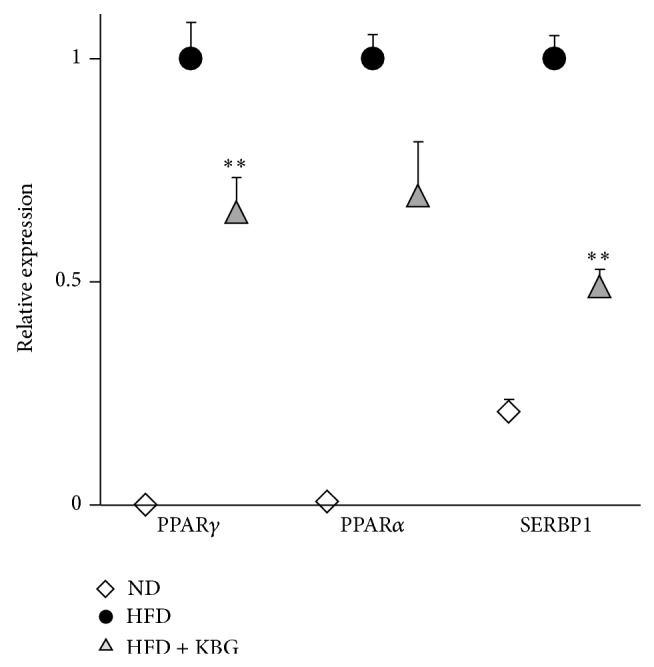
Effect of KBG on obesity-associated gene expression in liver tissue of DIO mice. C57BL/6 mice were fed with normal diet (ND) or high-fat diet (HFD) for 10 weeks and then administered with control water or KBG (500 mg/kg, p.o., daily) for 12 weeks under the same feeding condition. After 8 weeks of control or KBG treatment, mice fed with HFD were changed to ND until the termination of experiment with maintaining KBG treatment for another 4 weeks. Liver tissue samples were collected upon the termination and the relative expression of obesity-associated genes (PPARg, PPARa, and Srebf1) was determined by RT-PCR and shown as relative expression to HFD group (HFD = 1). Data are mean ± SEM (*n* = 7–15). ^**^ *P* < 0.001.

To further explore the therapeutic benefit of KBG in obesity, we examined the effect of KBG on the expression of liver lipids in both SHR rats and DIO mice. While KBG did not show any significant effect on liver content of TG, cholesterol, and FFA in the SHR rat (Figure 4(a)), the liver TG and cholesterol level (but not FFA level) of the KBG-treated DIO mice were significantly lower than those of the control group (Figure 4(b)). In concert with such a reduction in the liver lipid contents, we observed less adipocyte accumulation in the liver of DIO mice treated with KBG (Figure 5(a), upper panels) and indeed the steatosis score was significantly lower in DIO mice treated with KBG (Figure 5(b)). Furthermore, the size of adipocytes in white adipose tissue, which was enlarged in DIO mice, was relatively smaller with KBG treatment (Figure 5(a), lower panels) as seen in the increased number of adipocytes on histological examination (Figure 5(c)). We then examined the effect of KBG on the mRNA expression of molecules associated with lipid metabolism in liver to understand the potential molecular mechanism that underlies the control of lipid metabolism in KBG-treated DIO mice, by using quantitative real-time PCR. As shown in Figure 6, we found that the mRNA expressions of PPAR*γ* and SREBP1 in the liver were significantly decreased by KBG treatment in DIO mice.

## 4. Discussion

KBG is a traditional Kampo medicine that has been widely used for improving blood circulation and is also known for its anti-inflammatory or scavenging effect, hence its relevance to obesity being expected [8, 10]. In this study, we employed two distinct obesity preclinical animal models, driven by a genetic or dietary mechanism, to test the therapeutic efficacy of KGB. While KBG did not show any significant impact on body composition of either obesity model, the serum level of leptin and the liver TG content were significantly decreased by KGB treatment in the diet-induced obesity mouse model. Considering these results, KBG is presumably not actively effective in body composition in diet-induced obesity but rather effective in modulating the metabolic status of adipocytes or lipid metabolism. Indeed, lower liver lipid contents and lower liver fat deposition were observed in DIO mice treated with KGB. Furthermore, the size of adipocytes in white adipose tissue was much smaller in DIO mice treated with KBG. As such differences were not observed in DIO mice maintained on high-fat diet feeding conditions solely treated with KBG (data not shown), we speculate that the antiobesity effect of KBG may require the presence of diet therapy. Although the exact mechanism of the lipid-lowering effect of KBG in vivo has not been clarified yet, we observed the alteration in the liver mRNA expression of molecules associated with lipid metabolism (PPAR*γ* and SREBP1) in DIO mice treated with KGB [15–17]. Considering the result that KGB did not have an effect on much of those serum parameters and liver lipid contents in the leptin-deficient SHR rat model, we also presume that the lipid-lowering effect of KGB may be mediated by regulating the systemic leptin level. As leptin has been widely known as a potent lipid-lowering adipokine and considered as an important factor in preventing cellular lipotoxicity and insulin resistance [5, 18], the modulating effect of KBG or its active component in leptin may reside at least partially in the mechanism of action for the lipid-lowering effect of KBG. In this context, it would be important to further explore any correlation between the clinical response to KBG and leptin resistance in obesity patients.

## Figures and Tables

**Table 1:** Components of the keishibukuryogan formula.

Crude drugs	Weight ratio (g)
*Cinnamomi Cortex *	3.0
*Poria cocos *	3.0
*Moutan Cortex *	3.0
*Persicae Semen *	3.0
*Paeoniae Radix *	3.0

**Table 2:** Sequences of the primers used in real-time PCR of the mouse tissue.

Gene	Forward primer	Reverse primer
PPAR*γ*	GAACCTGCATCTCCACCTTATT	TGGAAGCCTGATGCTTTATCC
PPAR*α*	CGGTGTGTATGAAGCCATCT	TAAGGAACTCGCGTGTGATAAA
SREBP-1	CATCGACTACATCCGCTTCTT	CACCAGGTCCTTCAGTGATTT
